# Induction of penicillin tolerance during pregnancy: Allergological opinion on the recommendation of the current AWMF Guidelines on Diagnosis and Treatment of Syphilis (AWMF Registry No. 059-002) 

**DOI:** 10.5414/ALX02224E

**Published:** 2021-01-26

**Authors:** Bettina Wedi, Werner Aberer, Knut Brockow, Heinrich Dickel, Randolf Brehler, Thilo Jakob, Burkhard Kreft, Vera Mahler, Hans F. Merk, Norbert Mülleneisen, Hagen Ott, Wolfgang Pfützner, Stefani Röseler, Franziska Ruëff, Cord Sunderkötter, Axel Trautmann, Regina Treudler, Margitta Worm, Gerda Wurpts

**Affiliations:** 1 Department of Dermatology, Allergology, and Venereology, Comprehensive Allergy Center, Hannover Medical School, Germany,; 2 Department of Dermatology, Medical University Graz, Austria,; 3 Department and Outpatient Clinic for Dermatology and Allergology am Biederstein, Technical University of Munich,; 4 Department of Dermatology, Venereology, and Allergology, St. Josef-Hospital, University Hospital of the Ruhr University of Bochum,; 5 Department of Dermatology, University Hospital Muenster,; 6 Department of Dermatology and Allergology, University Medical Center Gießen and Marburg, Campus Gießen,; 7 Department of Dermatology and Venereology, University Hospital Halle (Saale),; 8 Paul-Ehrlich-Institut, Langen,; 9 Dermatologists at the Merckhaus Dr. Herbst and Kollegen, Darmstadt,; 10 Department of Dermatology and Allergology, Aachen Comprehensive Allergy Center (ACAC), University Hospital RWTH Aachen,; 11 Lung and Allergy Center, Leverkusen,; 12 Pediatric Dermatology and Allergology, Children’s and Youth Hospital “Auf der Bult”, Hannover,; 13 Department of Dermatology and Allergology, University Medical Center Gießen and Marburg, Campus Marburg,; 14 Augustinians Hospital, Academic Teaching Hospital of the University of Cologne,; 15 Department and Outpatient Clinic of Dermatology, and Allergology, Allergy Center, Hospital of the University of Munich,; 16 University Hospital and Outpatient Clinic of Dermatogy and Venereology, University Hospital Halle (Saale),; 17 Department and Outpatient Clinic of Dermatology, Venereology, and Allergology, Allergy Center Mainfranken, University Hospital Würzburg,; 18 Department and Outpatient Clinic of Dermatology, Venereology, and Allergology and Leipzig Interdisciplinary Allergy Center – LICA-CAC, University of Leipzig,; 19 Department for Dermatology, Venereology, and Allergology, Charité-University Medicine Berlin, Allergy Center-Charité (ACC), Berlin,; 20 Department of Dermatology and Allergology, Aachen Comprehensive Allergy Center (ACAC), University Hospital RWTH Aachen, Germany

**Keywords:** penicillin allergy, pregnancy, syphilis, desensitization, tolerance induction

## Abstract

Not available.

## Statement

The update of the S2k Guideline on Diagnosis and Therapy of Syphilis [1], AWMF (Arbeitsgemeinschaft der Wissenschaftlichen Medizinischen Fachgesellschaften e.V. (Association of the Scientific Medical Societies in Germany)) Registry No. 059-002, recommends to carry out specific immunotherapy in patients with penicillin allergy (penicillin desensitization), and strong consensus is indicated for this recommendation (12/12). 

As authors of the S2k Guideline on Diagnostic Procedures for Suspected Hypersensitivity to Beta-lactam antibiotics (AWMF Registry No. 061-032) [2] we would like to express our opinion. No specialist allergological society was involved in developing the syphilis guideline. 

We would like to point out that desensitization with a drug is generally, and therefore also during pregnancy, associated with a not inconsiderable risk, especially when the patient has a history of proven or highly probable IgE-mediated anaphylactic reaction. In the case of the much more frequent late-type reaction (exanthema), tolerance induction is rather not an option; although a severe reaction is not to be expected, hardly any data are available on the effectiveness of tolerance induction. 

The recommendation of the syphilis guideline uses the term “specific immunotherapy”, which should be reserved for allergen-specific immunotherapy (hyposensitization). The term desensitization is widespread, particularly in English-speaking countries, but it also implies that permanent tolerance can be achieved. It should be noted that, in contrast to specific immunotherapy, the induced tolerance to drugs only lasts for hours or a few days after the end of therapy. Tolerance induction would have to be repeated, if administration of the drug is required again after a while. 

A query among the authors of the Guideline on Diagnostic Procedures for Suspected Hypersensitivity to Beta-lactam antibiotics showed that, with one exception (years ago and with weak history-based evidence of a specific reaction as well as negative skin test), none of the so-far 15 responding university allergy facilities (eight of them certified as Comprehensive Allergy Center) has already carried out induction of penicillin tolerance in a pregnant woman. None of the authors considers induction of penicillin tolerance during pregnancy to be uncomplicated. Anaphylactic reactions during tolerance induction with increasing doses can never be completely ruled out; both severe anaphylaxis and the required treatment can cause serious complications for the mother and the fetus. 

**Figure 1:**
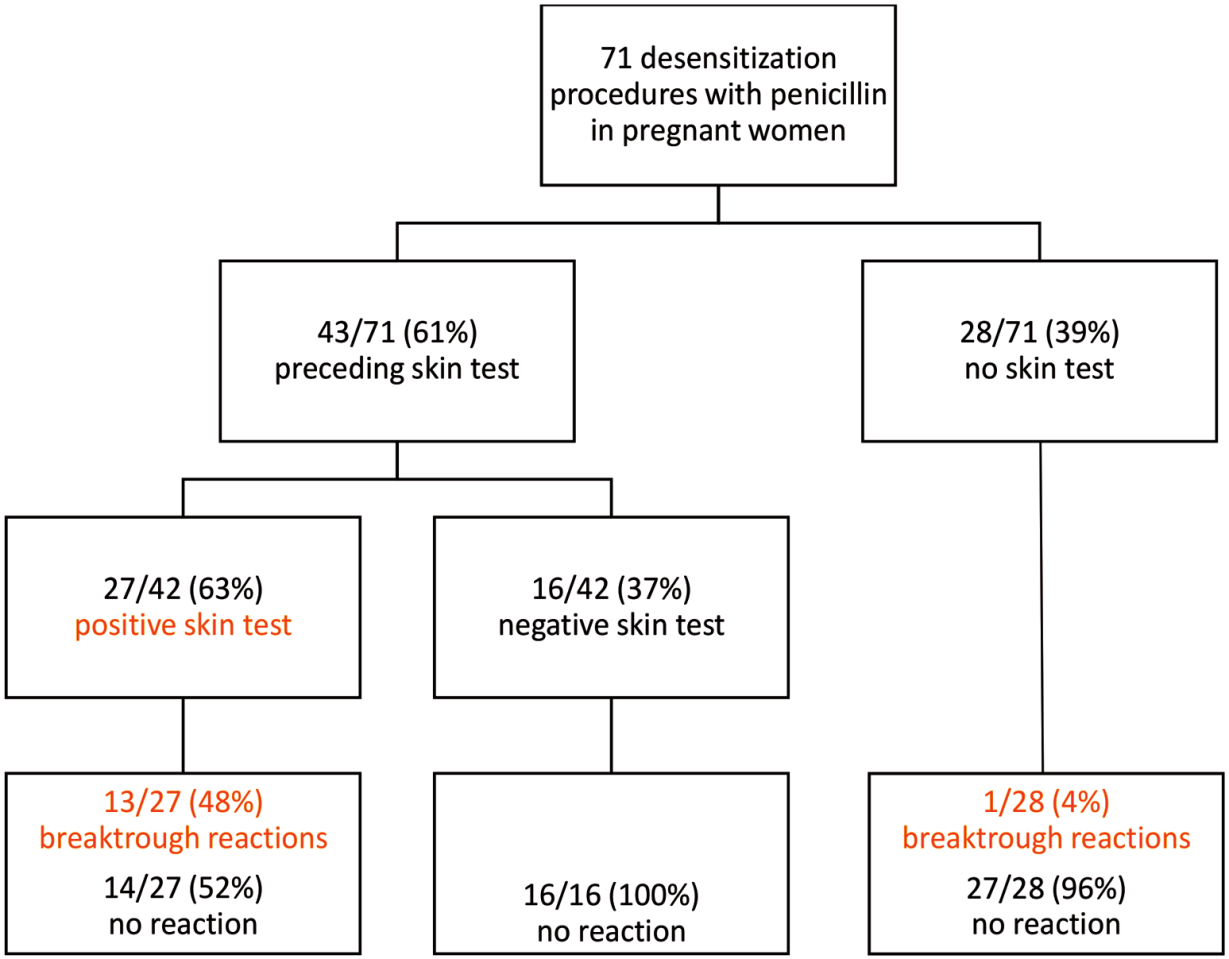
Penicillin desensitization procedures during pregnancy published in a current systematic review [3].

Successful tolerance induction in pregnant women with penicillin allergy and syphilis has been described in South America and the USA, and a current systematic review, which has been available online since the end of January 2020, summarizes these reports [3]. Of a total of 1,621 papers, the review included 18, of which 14 were not older than 20 years [3]. These were case reports and observational studies with low patient numbers and methodological deficiencies [3]. Details on penicillin allergy and the baseline sensitization status, i.e., results from previous diagnostic work-up, were frequently missing [3]. In some cases, only published abstracts [4, 5] but no full publications were available. In total, 71 tolerance inductions were described (Figure 1). In conclusion, induction of penicillin tolerance was tolerated by 57/71 (80%) of pregnant women (Figure 1). It should be noted, however, that 28 and 16, respectively, of the 71 had no previous skin test or the skin test was negative and these pregnant women were desensitized solely based on their patient history or a positive specific IgE result so that a penicillin allergy could not be reliably demonstrated. Of the 27 pregnant women with positive penicillin skin test, almost half (48%) had a reaction during tolerance induction (Figure 1) [3], including anaphylaxis (n = 2), pruritus (n = 4), urticaria (n = 3), and other, non-specified conditions (n = 4) [3]. In 1 case, a severe anaphylactic reaction with uterine contractions was observed [4]. An article published in 1985 describes a pregnant woman with listeriosis; during tolerance induction she experienced two episodes of urticaria with later development of cramps and intermittent vaginal bleedings for 11 days, followed by spontaneous abortion [6]. In general, however, the 13 tolerance inductions carried out in pregnant women with syphilis and described in this older article [6] were well tolerated by mothers and fetuses. 

The S2k Guideline on Diagnostic Procedures for Suspected Hypersensitivity to Beta-lactam antibiotics [2] states the following: 

*In the case of a history of immediate reactions or proven allergy to a BLA and urgently indicated use of the suspected BLA or a BLA with a high risk of cross-reactivity, desensitization needs to be considered (see Sect. “Desensitization (tolerance induction)”) after a decision has been taken on the individual case.*

The following recommendation can be found in the section “Desensitization”: 

*Desensitization should be considered as an option if a drug is required in patients with proven or highly likely immediate allergy and no alternative treatment is available or satisfactory. A positive benefit–risk assessment is required.*

For mild late-type reactions such as maculopapular or fixed drug eruptions, the success of tolerance induction is controversial, and desensitization is even contraindicated in patients with type II and III reactions according to the Gell and Coombs classification and severe late-type reactions such as SJS/TEN and DRESS/DIHS [2]. 

The Guideline on Diagnostic Procedures for Suspected Hypersensitivity to Beta-lactam antibiotics [2] does not refer to pregnant women. In the Guideline for the Diagnosis of Drug Hypersensitivity Reactions (AWMF Registry No. 061-021), pregnancy is listed as a contraindication for challenge testing [7]. 

In the long version of the S3 guideline on patch testing [8], although there are no proven negative effects, performing a patch test during pregnancy and breastfeeding is not recommended for general safety reasons (low evidence, but strong consensus of 100%). 

According to the 2010 European Consensus Recommendations on tolerance induction in immediate-type drug hypersensitivity reactions [9], tolerance induction is indicated if: (1) the drug in question is indispensable (here the example of penicillin in pregnant women is given) and (2) the drug in question is more effective than available alternatives. 

In this respect, the question arises whether penicillin can be replaced by other drugs during pregnancy and whether these are just as effective for syphilis. Penicillin is one of the antibiotics of choice during pregnancy. According to Embryotox [10], with a high level of experience with several thousand evaluated pregnancies, there was no evidence of an increased risk of malformations when penicillin was used, and previous observations speak against a fetotoxic risk. Also for cephalosporins, there was no evidence of an increased risk of malformations or a fetotoxic risk based on moderate experience with several hundred evaluated pregnancies [10]. In small case studies, ceftriaxone was successfully used for the treatment of syphilis during pregnancy with an effect comparable to that of penicillin. According to the product information, ceftriaxone should be prescribed to pregnant women, especially in the first 3 months, only if the therapeutic benefits outweigh the risks. According to Embryotox [10], ceftriaxone can be used during pregnancy if the spectrum of pathogens requires it. 

In addition to the dilemma of potentially risky in-vivo diagnosis, the problem illustrates the dilemma of treating pregnant women with drugs that have not been explicitly approved for this. Penicillin has been approved for use during pregnancy but is contraindicated in cases of proven penicillin hypersensitivity. The use of approved therapeutic drugs for diagnostic purposes or of penicillin for desensitization purposes in appropriate dosing schemes is an off-label use, which among other things results in special requirements for pre‐treatment consultation. Cross-reactivity between penicillins and cephalosporins like cefuroxime and ceftriaxone is very rare [2]. A negative intradermal test with cephalosporins is also a good predictor for good tolerability [11]. Studies on cephalosporins used as a first preparation in pregnant women with syphilis are desirable, but cannot realistically be expected. 

A query among the authors of the Guideline on Diagnostic Procedures for Suspected Hypersensitivity to Beta-lactam antibiotics showed that they would prefer using ceftriaxone for syphilis therapy during pregnancy over carrying out penicillin tolerance induction as long as the patient has negative prick test and intradermal test results for ceftriaxone. 

In summary, we would like to critically question or weaken the strong recommendation for an induction of penicillin tolerance in pregnant women (should be carried out, consensus 12/12). From an allergological point of view, it can only be considered in individual cases, after carefully weighing the benefits and individual risks. Ceftriaxone is an effective alternative, and cross-reactions with penicillin G or V and the aminopenicillins are very unlikely, especially if the intradermal test is negative. From a microbiological point of view, ceftriaxone has the disadvantage, compared to penicillin, that it exerts a relatively high resistance selection pressure on the gastrointestinal microbiome (e.g., because of its significantly broader spectrum of activity compared to penicillin, which also includes many Gram-negative bacteria, and its relatively high hepatobiliary excretion). The current version of the syphilis guideline explicitly discusses ceftriaxone as an alternative, but elsewhere. In the recommendation on tolerance induction to penicillin, this alternative is not considered. 

In analogy to our general recommendations for tolerance induction in the Guideline on Diagnostic Procedures for Suspected Hypersensitivity to Beta-lactam antibiotics, we suggest the following recommendation for the course of action in the case of a history of penicillin allergy in pregnant women with syphilis:

**The possibility of induction of penicillin tolerance in a pregnant woman with syphilis can be considered if penicillin is considered urgently necessary despite a proven or highly probable immediate-type allergy and an alternative, such as ceftriaxone, is not available or is not considered to be satisfactorily effective. The benefit of penicillin and the individual risk of an anaphylactic reaction during tolerance induction must be carefully weighed.**

All this also prompts us to urgently appeal to all doctors, to only label children, adolescents, or young women as having “penicillin allergy” if there is justified suspicion and then to have this checked up promptly, before conception. 

## Acknowledgment 

We thank Professor Dr. Alexander Kapp, who was the first to point out the discrepancy between the recommendations. 

## Funding 

No funding was received to assist with the preparation of this manuscript. 

## Conflict of interest 

The authors indicate that there is no conflict of interest related to this paper. Vera Mahler: The content and positions expressed in this statement reflect the personal expert opinion of the author and cannot be interpreted or quoted as if they had been expressed by or given on behalf of the competent national higher federal authority, the European Medicines Agency or one of its committees or working groups or would reflect their position.
